# PReS-FINAL-2309: Juvenile systemic lupus erythematosus: a case series depiction in an urban community and a comparison to an adult case series

**DOI:** 10.1186/1546-0096-11-S2-P299

**Published:** 2013-12-05

**Authors:** V Torrente-Segarra, E Iglesias, MI Gonzalez, J Sanchez, S Ricart, R Bou, T Salman-Monte, J Carbonell, J Antón-López

**Affiliations:** 1 Pediatric Rheumatology, Hospital Sant Joan Deu, Esplugues Llobregat, Spain; 2 Rheumatology, Hospital del Mar, Barcelona, Spain

## Introduction

Clinical features comparison between adut and pediatric SLE patients.

## Objectives

to describe clinical and serological features of juvenile Systemic Lupus Erythematosus (jSLE) patients; to compare the main differences between jSLE characteristics and adult SLE (aSLE).

## Methods

we detected all our jSLE patients from our database. We collected sociodemographic data and both clinical and serological variables from our jSLE patients' charts at Hospital Sant Joan de Déu, Esplugues (Catalunya, Spain). We defined the following variables: cutaneous disease (as presence of discoid lupus, photosensitivity, and/or malar rash), joint disease (arthritis), hematological disease (anemia, leucopenia, and/or plaquetopenia), renal disease (>0.5 g/d proteinuria and, if available, histological WHO class), neurolupus (psychosis and/or convulsions). We collected the following data: age at onset, time disease evolution, and gender. In regarding to serological markers: DNAds positivity through follow-up was recorded. We also collected information from a well-recognised aSLE cohort of 124 patients in the same Mediterranean urban area. We analysed all data in order to depict the type of clinical and serological features for each group of patients.

## Results

we assessed charts from 42 jSLE (n = 42), and compared to aSLE (n = 124). 90% of the jSLE patients were female, compared to a 95% of the aSLE cohort. Age at onset was 12.1 years in jSLE. In the jSLE group of patients: 81% had had cutaneous disease, 62% haematological disorder, 44% arthritis, 40% nephropathy (60% class IV, 20% class III, 10% class II and 10% class V), and 14% convulsions. In the aSLE cohort: 80% had cutaneous disease, 54% haematological disorder, 29% arthritis, 14% nephropathy and 3.2% neuro-lupus. DNAds positivity was 68% in jSLE and 54.8% in aSLE.

## Conclusion

jSLE and aSLE are slightly different in our Mediterranean region. Most of cases were women and main features were similar in both groups. Pediatric patients had more frequently nephropathy (most of them class IV-WHO), and DNAds positivity. Further follow-up, in which are already involved, is needed to assess the outcome of our jSLE.

## Disclosure of interest

None declared.

